# Intravenous Neuromyelitis Optica Autoantibody in Mice Targets Aquaporin-4 in Peripheral Organs and Area Postrema

**DOI:** 10.1371/journal.pone.0027412

**Published:** 2011-11-04

**Authors:** Julien Ratelade, Jeffrey L. Bennett, A. S. Verkman

**Affiliations:** 1 Departments of Medicine and Physiology, University of California San Francisco, San Francisco, California, United States of America; 2 Departments of Neurology and Ophthalmology, University of Colorado Denver, Aurora, Colorado, United States of America; The University of Hong Kong, Hong Kong

## Abstract

The pathogenesis of neuromyelitis optica (NMO) involves binding of IgG autoantibodies (NMO-IgG) to aquaporin-4 (AQP4) on astrocytes in the central nervous system (CNS). We studied the *in vivo* processing in mice of a recombinant monoclonal human NMO-IgG that binds strongly to mouse AQP4. Following intravenous administration, serum [NMO-IgG] decreased with t_1/2_ ∼18 hours in wildtype mice and ∼41 hours in AQP4 knockout mice. NMO-IgG was localized to AQP4-expressing cell membranes in kidney (collecting duct), skeletal muscle, trachea (epithelial cells) and stomach (parietal cells). NMO-IgG was seen on astrocytes in the area postrema in brain, but not elsewhere in brain, spinal cord, optic nerve or retina. Intravenously administered NMO-IgG was also seen in brain following mechanical disruption of the blood-brain barrier. Selective cellular localization was not found for control (non-NMO) IgG, or for NMO-IgG in AQP4 knockout mice. NMO-IgG injected directly into brain parenchyma diffused over an area of ∼5 mm^2^ over 24 hours and targeted astrocyte foot-processes. Our data establish NMO-IgG pharmacokinetics and tissue distribution in mice. The rapid access of serum NMO-IgG to AQP4 in peripheral organs but not the CNS indicates that restricted antibody access cannot account for the absence of NMO pathology in peripheral organs.

## Introduction

Neuromyelitis optica (NMO) is an autoimmune inflammatory disease of the central nervous system (CNS) associated with demyelinating lesions mainly in optic nerve and spinal cord, leading to blindness and paralysis [1], [2]. The majority of NMO patients are seropositive for autoantibodies (NMO-IgG) against extracellular epitope(s) on aquaporin-4 (AQP4) [3], [4], [5], a plasma membrane water channel expressed in astrocytes throughout the CNS [6], [7]. AQP4 is also expressed in cell plasma membranes in various peripheral tissues, including kidney collecting duct, skeletal muscle, gastric parietal cells, tracheal epithelial cells, airway epithelium and exocrine gland epithelium [6], [8]. Mice deficient in AQP4 do not manifest significant baseline abnormalities in the CNS, but show, under appropriate stresses, impairment in brain and spinal cord water balance, neuroexcitation and glial scar formation [9]. AQP4-deficient mice do not manifest significant peripheral abnormalities, such as skeletal muscle dysfunction [10] or reduced gastric acid secretion [11], except for a very mild impairment in maximal urinary concentrating ability [12].

Indirect evidence suggesting that serum NMO-IgG is pathogenic includes the high specificity of NMO-IgG seropositivity for NMO [1], correlations between serum NMO-IgG titers and disease activity [13], [14], loss of AQP4 in NMO lesions [15], and the clinical benefit of NMO-IgG depletion by plasma exchange [16]. There is considerable interest in the development of animal models of NMO for elucidation of the mechanisms of NMO disease pathogenesis and for testing of candidate therapies. In rats with pre-existing neuroinflammation, as produced in models of experimental autoimmune encephalomyelitis, peripheral NMO-IgG or recombinant AQP4 antibody administration produces neuroinflammatory lesions [17], [18], [19]. In naïve mice, intracerebral administration of NMO-IgG with human complement produces lesions with NMO-like characteristics including inflammation, loss of AQP4 and GFAP immunoreactivity, perivascular deposition of activated complement, and myelin loss [20]. NMO-IgG binding to AQP4 in astrocytes is thought to initiate a cascade of inflammatory events, including antibody-dependent complement and cell-mediated astrocyte damage, leukocyte recruitment, cytokine release and demyelination [1], [21], [22].

Though the rodent data suggest a pathogenic role of NMO-IgG in NMO, they involve non-physiological maneuvers such as induction of pre-existing neuroinflammation or direct infusion of NMO-IgG and complement into brain. Key unresolved issues in NMO pathogenesis include the preponderance of NMO lesions in optic nerve and spinal cord over brain and peripheral AQP4-expressing tissues, and the mechanisms by which NMO-IgG in the blood enters the CNS to initiate disease.

To test the hypothesis that restricted access of serum NMO-IgG to AQP4 in peripheral tissues might account for the absence of NMO pathology in the periphery, and as a first step in generating mouse models of NMO based on peripheral NMO-IgG administration, we determined the cellular distribution and pharmacokinetics of NMO-IgG following peripheral and CNS administration. For these studies we used a recombinant monoclonal human NMO-IgG antibody (rAb-53) that was characterized previously [17], [23] and found here to bind strongly to mouse AQP4. The use of purified monoclonal human NMO-IgG allowed the sensitive and unambiguous determination of antibody localization and serum concentration in mice, which is not possible using human NMO serum or purified IgG in which NMO-IgG comprises a very small fraction of total IgG.

## Results

### Human monoclonal NMO-IgG antibody rAb-53 binds to mouse AQP4

A series of monoclonal recombinant antibodies was previously generated by single-cell PCR done on individual plasmablasts from cerebrospinal fluid of NMO patients [17]. We found that recombinant antibody rAb-53, which strongly binds to human AQP4 [23], bound well to mouse AQP4 and caused NMO-like lesions following intracerebral administration (data not shown). AQP4 is expressed in astrocytes as well as in other tissues as two major isoforms resulting from alternative transcripts with translation initiation at Met-1 (M1) and Met-23 (M23) [24], [25]. M23-AQP4 forms orthogonal arrays of particles (OAPs), which are membrane aggregates that assemble in regular arrays, whereas M1-AQP4 does not [26]. Previous studies have shown that OAPs are the preferential target of NMO-IgG [23], [27].

**Figure 1:**
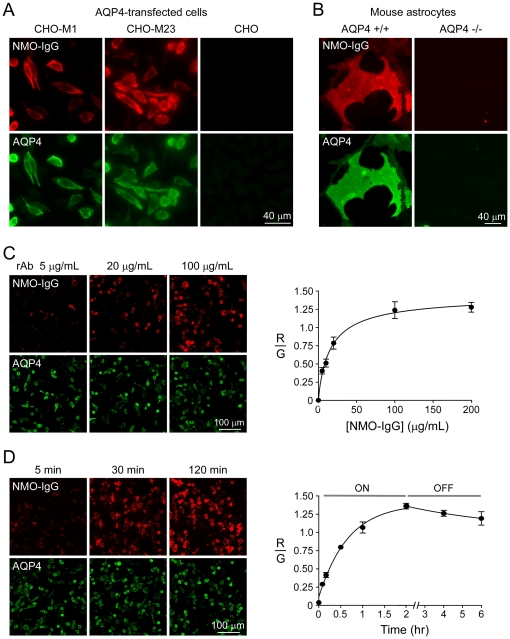
Binding of a recombinant monoclonal NMO-IgG to mouse AQP4. A. CHO cells expressing mouse M1 or M23 AQP4 were labeled with an anti-AQP4 C-terminus antibody (green) and recombinant monoclonal NMO antibody rAb-53 (labeled ‘NMO-IgG’, red). B. Binding of NMO-IgG to primary astrocyte cultures from wild type (+/+) and AQP4 null (-/-) mouse brain. C. (left) Fluorescence micrographs of NMO-IgG (red) binding to mouse M23-AQP4 as a function of concentration. AQP4 labeled with AQP4 antibody (green). (right) NMO-IgG binding to mouse M23-AQP4 shown as ratio of red (NMO-IgG) to green (reference AQP4) fluorescence (mean±S.E., n = 10). Curve is fit to a single-site binding model. D. Binding kinetics of NMO-IgG (rAb-53, 50 µg/ml, red) to mouse M23-AQP4 (green). Unbinding measured following 120 min incubation and washout for indicated times (mean±S.E., n = 10). Data are representative of 2 independent sets of experiments.

Fig. 1A shows staining of CHO cells that were transiently transfected with the M1 or M23 isoforms of mouse AQP4. NMO-IgG (rAb-53) bound well to both the M1 and M23 isoforms (Fig. 1A), as well as to primary cultures of astrocytes from neonatal mouse brain (Fig. 1B), in which both the M1 and M23 AQP4 isoforms are present and coassemble in heterotetramers that form OAPs [28]. Staining was not seen in non-transfected CHO cells (Fig. 1A) or in astrocyte cultures from AQP4 knockout mice (Fig. 1B). Fluorescence micrographs in Fig. 1C show concentration-dependent binding of rAb-53 to mouse M23-AQP4. As quantified by the ratio of red (NMO-IgG) to green (AQP4) fluorescence (Fig. 1C), antibody binding fitted well to a single-site binding model, as found previously with human M23-AQP4 [23]. The apparent dissociation constant for rAb-53 binding to mouse M23-AQP4 was 111±39 nM. Fig. 1D shows kinetics of rAb-53 binding to mouse M23-AQP4 after incubation with rAb-53 and after washout. NMO-IgG binding occurs over tens of minutes and washout over many hours.

### NMO-IgG pharmacokinetics

**Figure 2:**
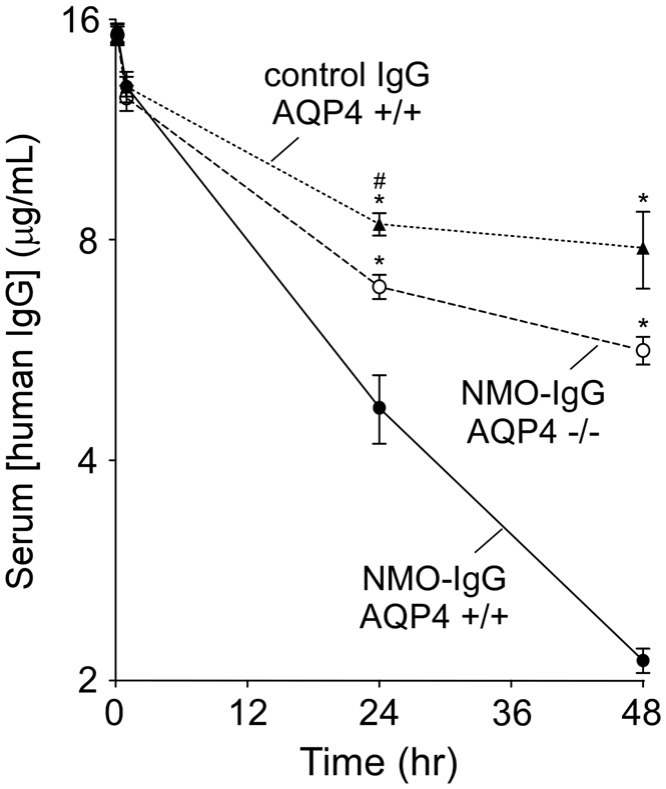
Serum pharmacokinetics of NMO-IgG. Serum antibody concentration (NMO-IgG and control IgG) in wild type (+/+) and AQP4 knockout (-/-) mice quantified by ELISA at 10 min, and 1, 24 and 48 hours after intravenous injection. Data are expressed in µg/mL and plotted on a log_2_ scale (mean±S.E., 4 mice per group). * *P*<0.05 compared with NMO-IgG +/+. #*P*<0.05 compared with NMO-IgG -/-.

The pharmacokinetics of NMO-IgG in mice was determined from the time course of serum concentration following intravenous administration of 20 µg antibody by tail vein. As a human IgG1 antibody, NMO-IgG concentration in mouse serum was assayed by ELISA, without interference by the great excess of mouse IgG. Fig. 2 shows decreasing NMO-IgG concentration over 48 hours following injection, with t_1/2_ ∼18 hours. To determine whether binding to AQP4 facilitated NMO-IgG clearance from blood, parallel studies were done in weight-matched AQP4 knockout mice. AQP4 deletion significantly slowed the disappearance of NMO-IgG, with t_1/2_ ∼41 hours (Fig. 2), suggesting that binding of NMO-IgG to AQP4-expressing tissues such as skeletal muscle in wild type mice reduces its serum concentration. In addition, a control human IgG1 antibody that does not target any mouse antigen had a relatively long t_1/2_ ∼58 hours in wild type mice. These t_1/2_ values are in the range of those found for human IgGs in mice [29].

### In vivo targeting of NMO-IgG to AQP4-expressing tissues

**Figure 3:**
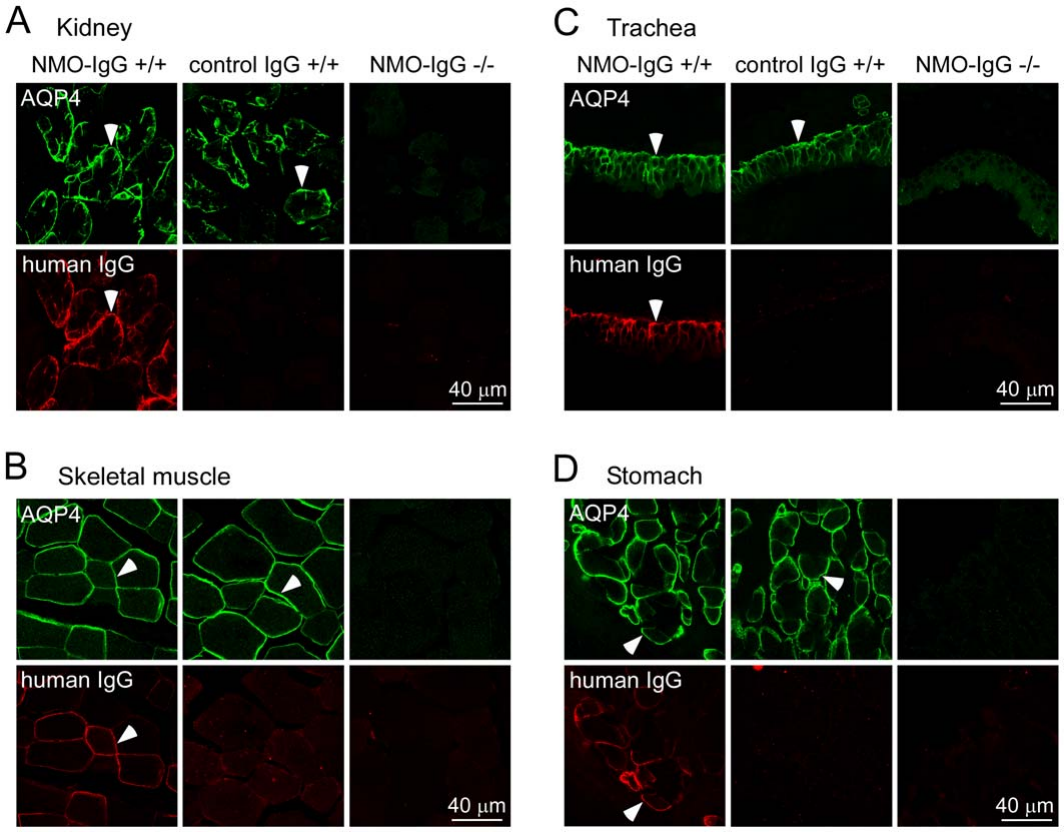
NMO-IgG localization to AQP4-expressing peripheral organs following intravenous administration. NMO-IgG or a control human IgG were injected intravenously in wild type and AQP4 knockout mice. After 24 hours, tissues were fixed and stained for AQP4 (green). NMO-IgG and control IgG were detected using a secondary anti-human antibody (red). Confocal fluorescence images shown of kidney (A), skeletal muscle (B), trachea (C) and stomach (D). Arrowheads indicate basolateral membrane of kidney collecting duct (A), skeletal muscle sarcolemma (B), basolateral membrane of tracheal surface epithelial cells (C), and basolateral membrane of gastric parietal cells (D). Data are representative of 3 mice per group.

The tissue distribution of NMO-IgG was determined at 1 and 24 hours after intravenous administration, in which tissues were fixed and immunostained for AQP4 using an anti-AQP4 antibody and for NMO-IgG using an anti-human IgG fluorescent secondary antibody. NMO-IgG localization was studied in the major AQP4-expressing peripheral organs, including kidney, skeletal muscle, trachea and stomach. Studies were done in wild type and AQP4 knockout mice, as well as using a control (non-NMO) IgG in place of NMO-IgG. As shown in Fig. 3, AQP4 expression was found in wild type mice on the basolateral membrane of kidney collecting duct (panel A), the plasmalemma of fast-twitch fibers in skeletal muscle (panel B), the basolateral membrane of tracheal surface epithelial cells (panel C) and the basolateral membrane of gastric parietal epithelial cells (panel D). Immunostaining was negative in all tissues from AQP4 knockout mice. NMO-IgG colocalized with AQP4 in each of the tissues from wild type mice. Interestingly, there was some heterogeneity in NMO-IgG binding to AQP4-expressing cells (Fig. 3B), which may be due to variable accessibility of the antibody. NMO-IgG was not detected in tissues from AQP4 knockout mice, nor was human IgG detected in wild type mice following injection of control (non-NMO) IgG. Similar NMO-IgG localization was found in tissues from 3 mice studied at 24 hours after injection and 2 mice studied at 1 hour after injection (not shown), albeit with mildly reduced fluorescence.

**Figure 4:**
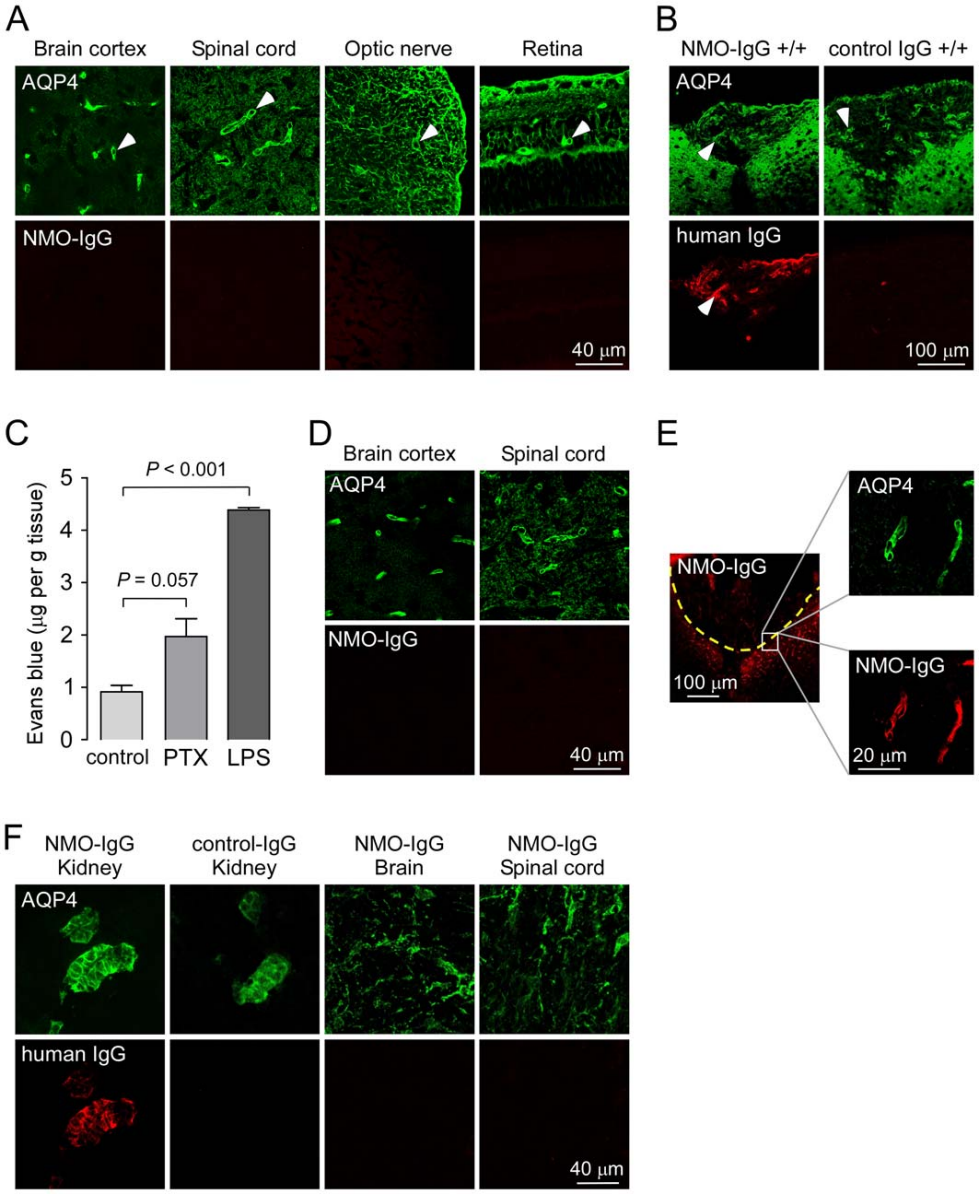
NMO-IgG localization in AQP4-expressing cells in the central nervous system following intravenous administration. A. Confocal fluorescence images for studies as in Fig. 3, showing AQP4 (green) and NMO-IgG (red) in retina, optic nerve, spinal cord and brain. B. Binding of NMO-IgG in the area postrema in brain. C. Evans blue quantification in brain of control mice and mice treated with pertussis toxin (PTX) or LPS (mean±S.E., 4 mice per group). D. Confocal fluorescence images showing AQP4 (green) and NMO-IgG (red) in brain and spinal cord of mice treated with LPS. E. Intravenously injected NMO-IgG enters brain parenchyma near the site of mechanical injury (dashed line, left panel) and binds to AQP4 on astrocyte end-feet around blood vessels (right panels). Data are representative of 4 mice. F. Pregnant mice (3 mice) were injected intravenously with NMO-IgG or control IgG. After 24 hours, embryos were fixed and stained for AQP4 (green) and NMO-IgG or control IgG (anti-human antibody, red).

Immunofluorescence was also done in AQP4-expressing tissues of the CNS from the same mice. AQP4 expression was seen on astrocytes in optic nerve, spinal cord and brain as well as on retinal Muller cells (Fig. 4A). However, except for the few restricted areas described below, NMO-IgG was not detected in these tissues following intravenous administration, despite a thorough examination of different regions of brain and spinal cord. Since NMO lesions have been reported in the area postrema of a few NMO patients [30], we labeled circumventricular organs, which lack an intact blood-brain barrier, by tail vein injection with Evans Blue. Remarkably, strong NMO-IgG binding to AQP4 was observed in the area postrema (Fig. 4B) as well as in the arcuate hypothalamic nucleus (not shown) following intravenous administration. No binding of a control IgG was detected in these areas (Fig. 4B).

### Attempts to enhance delivery of circulating NMO-IgG to the CNS

We tested maneuvers known to permeabilize the blood-brain barrier in an attempt to target intravenously administered NMO-IgG to AQP4 in brain and spinal cord. Pertussis toxin is known to permeabilize the blood-brain barrier and hence used to potentiate experimental allergic encephalomyelitis (EAE) in mice [31]. LPS has also been used to permeabilize the blood-brain barrier [32]. Pertussis toxin and LPS treatments increased the permeability of the blood-brain barrier to Evans blue by ∼2 and 4 fold, respectively (Fig. 4C). However, no NMO-IgG was detected in brain and spinal cord following LPS (Fig. 4D) or pertussis toxin (data not shown) administration. As a positive control, we found serum NMO-IgG in brain following mechanical damage to the blood-brain barrier produced by injuring superficial brain with a high-speed drill. Fig. 4E shows NMO-IgG binding in brain cortex near the site of mechanical injury (left panel). NMO-IgG colocalized with AQP4 at the end-feet of astrocytes in a perivascular pattern (Fig. 4E, right panels). We also investigated if NMO-IgG could access the developing central nervous system in which the blood-brain barrier may be more permissive [33]. Pregnant mice (embryonic stage E18-E19) were injected with 50 µg NMO-IgG (or control IgG) by the tail vein. After 24 hours, fetal organs were stained for NMO-IgG and AQP4 (Fig. 4F). At this stage, AQP4 is expressed in kidney and showed NMO-IgG localization (Fig. 4F), indicating passage of NMO-IgG between the maternal and fetal circulations. However, NMO-IgG was not detected in fetal brain or spinal cord, despite demonstrable AQP4 expression (Fig. 4F).

### Diffusion and binding of NMO-IgG in brain following intracerebral injection

**Figure 5:**
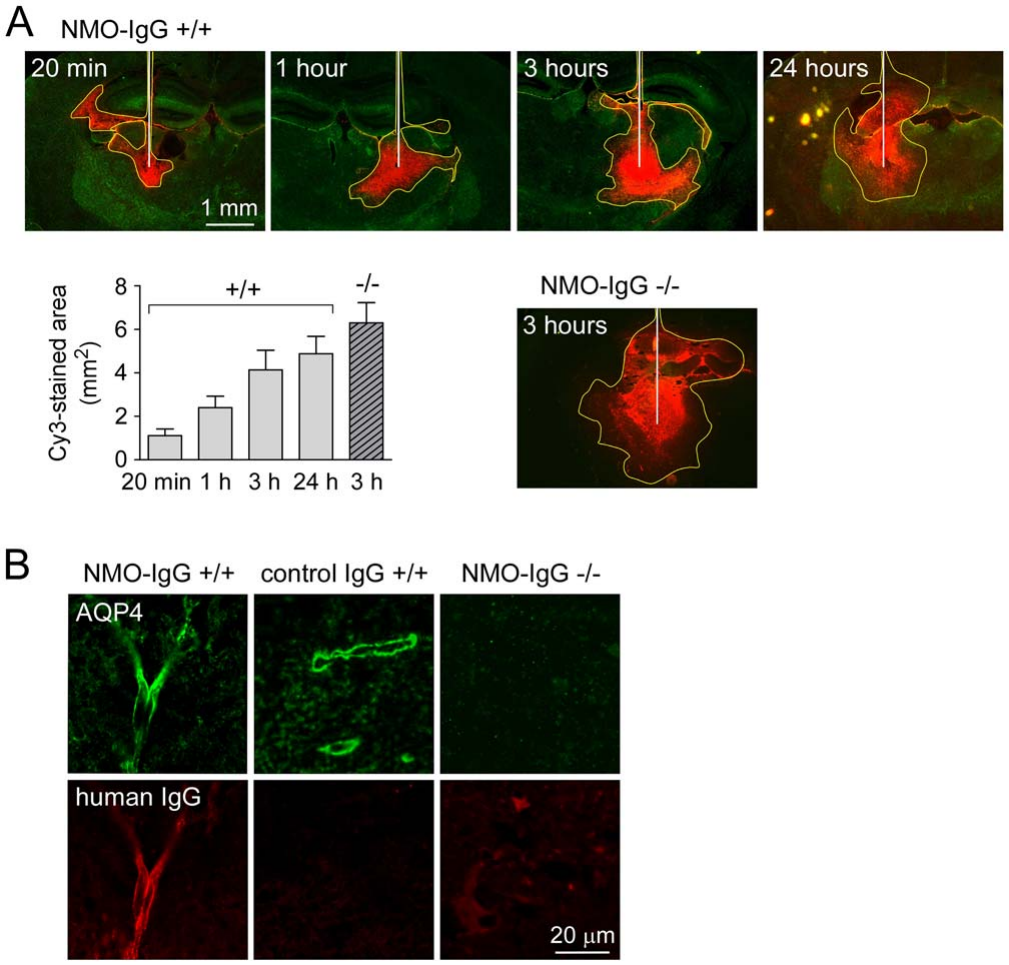
Binding and diffusion of NMO-IgG in mouse brain. A. Fluorescence micrographs showing distribution pattern of Cy3-labeled NMO-IgG at indicated times after direct injection into brain parenchyma in wild type (+/+) or AQP4 null mice (-/-). White line, needle track; yellow dashed line, area of NMO-IgG diffusion. Sections were also stained for AQP4 (green). The graph summarizes areas of Cy3 fluorescence (mm^2^, mean±S.E., 3 mice per group). B. Confocal fluorescence images showing colocalization of NMO-IgG (red) with AQP4 (green) in a perivascular pattern (3 h after injection).

The fate of NMO-IgG in brain tissue was investigated by fluorescence imaging of brain sections at different times after direct intra-parenchymal injection for Cy3-labeled NMO-IgG. We reasoned that NMO-IgG localization would depend on its diffusion in brain extracellular space and binding to AQP4 on astrocytes. Low magnification imaging in Fig. 5A shows progressively increasing areas of Cy3 fluorescence over time. The graph in Fig. 5A summarizes the spread of fluorescence as a function of time, showing an area of Cy3 fluorescence of 4.8 mm^2^ at 24 hours in wild type mice. The area was 4.1 mm^2^ at 3 hours, suggesting that the NMO-IgG was bound to AQP4 and thus restricted, as supported by an increased area of Cy3 fluorescence in mice lacking AQP4 (6.2 mm^2^). High magnification confocal imaging showed localization of injected NMO-IgG to astrocyte foot processes around vessels, which colocalized with AQP4 expression (Fig. 5B). Selective localization was not seen following injection of NMO-IgG in brain of AQP4 knockout mice or injection of control (non-NMO) IgG in brain of wild type mice (Fig. 5B).

## Discussion

In human NMO the concentration of NMO-IgG in serum is five hundred-fold or greater than that in cerebrospinal fluid [14]. How and when serum NMO-IgG enters the CNS to cause neuroinflammation and demyelination is unclear, as is why peripheral, AQP4-expressing organs are not affected in NMO. Our study addressed several questions: *(a)* does serum NMO-IgG access AQP4 in peripheral and central organs?; *(b)* under what conditions can serum NMO-IgG enter the CNS?; and *(c)* once in the CNS, over what area and at what rate does NMO-IgG diffuse? To address these questions we administrated a recombinant human monoclonal antibody in mice, either intravenously or directly into brain parenchyma, using as key controls an isotype-matched control human recombinant antibody, and mice lacking AQP4. In addition to addressing the NMO pathogenesis-relevant questions, our studies provide the first information on NMO-IgG pharmacology and tissue distribution for development of NMO mouse models.

We found that intravenously administered NMO-IgG was cleared slowly from serum in an AQP4-dependent manner, and rapidly accessed AQP4 in cell membranes in peripheral organs where AQP4 is expressed, including kidney, skeletal muscle, stomach and trachea. The relatively unrestricted access of NMO-IgG to AQP4 in peripheral tissues is a consequence of IgG diffusion across the leaky capillary endothelial barrier and binding to extracellular epitopes on cell surface-expressed AQP4. Selective AQP4 localization was not seen for control antibody or in AQP4 knockout mice, indicating the specificity of the NMO-IgG for AQP4. The ready access of NMO-IgG to AQP4-containing cells in the periphery indicates that restricted access cannot account for the absence of NMO pathology in non-CNS tissues. Therefore, other mechanisms to account for the resistance of peripheral tissues to NMO-IgG damage, such as differences in astrocyte vs. peripheral cell biology, mandate consideration.

Though intravenously administrated NMO-IgG was readily visualized in AQP4-expressing cells throughout the periphery, with one exception it was undetectable throughout CNS tissues including brain, spinal cord, optic nerve and retina, despite the sensitivity and specificity of the anti-human fluorescent secondary antibody. However, strong binding of NMO-IgG to AQP4 in brain was found in circumventricular organs such as area postrema, which contain fenestrated endothelial cells that allow diffusion from blood into brain tissue [34]. Interestingly, NMO-like lesions have recently been reported in the area postrema in some NMO patients with nausea and vomiting at clinical presentation [30]. These restricted lesions are characterized by a loss of AQP4 expression, inflammation and perivascular complement deposition. Our finding provides a potential explanation for the pathogenesis of these lesions. The permissive blood-brain barrier in the area postrema allows binding of NMO-IgG to AQP4, resulting in astrocyte pathology and chemosensory dysfunction. Indeed, it has been shown in cell culture *in vitro* and in brain *in vivo* that NMO-IgG activates complement, leading to astrocyte cytotoxicity, which accounts for the loss of AQP4, GFAP and complement deposition in NMO lesions [20], [35]. It has also been demonstrated *in vitro* that NMO-IgG can damage astrocytes in a complement-independent manner by recruiting natural killer cells [17], [36]. Primary astrocyte injury and initiation of an inflammatory cascade are believed to secondarily damage oligodendrocytes and neurons. However, for the studies here we did not expect NMO lesions in the area postrema because a human NMO recombinant antibody was used. It has been shown that intracerebral injection of purified IgG from serum of NMO patients causes lesions only when coinjected with human complement and not with mouse complement [20]. Engineering chimeric recombinant NMO antibodies, containing a mouse Fc portion, may be useful in follow-on work attempting to produce pathological lesions following peripheral antibody administration.

Various maneuvers to transiently permeabilize the blood-brain barrier, including peripheral pertussis toxin and LPS, did not result in detectable NMO-IgG deposition in CNS tissues. These maneuvers probably do not permeabilize the blood-brain barrier enough to allow robust passage of large macromolecules such as antibodies, despite increased permeability to Evans Blue. Other maneuvers causing greater blood-brain barrier permeabilization and non-specific systemic inflammation may allow more efficient antibody entry. For example, a recent study reported astrocyte damage after passive transfer of purified IgG from serum of NMO patients to rats immunized with complete Freund’s adjuvant (CFA) [37]. The authors demonstrate that CFA is sufficient to permeabilize the blood-brain barrier and allow entry of NMO-IgG into the CNS, although perivascular loss of AQP4 and GFAP was observed with IgG from only one out of three NMO patients [37]. Localization of intravenously administered NMO-IgG in brain was seen only after mechanical disruption of the blood-brain barrier. These data highlight the unresolved issue of how NMO-IgG enters the CNS to produce pathology especially in spinal cord and optic nerve. Perhaps small amounts of NMO-IgG, which are below the detection limit here, enter the CNS spontaneously or following a systemic inflammatory challenge. Alternatively, intrathecal plasmablasts producing anti-AQP4 autoantibodies may initiate CNS lesion formation resulting in initial blood-brain barrier breakdown through astrocyte destruction and CNS inflammation [17]. Plasma cells have been reported in both the CSF and tissue of NMO patients [17], [30], [38]. Following initial blood-brain barrier breakdown, continued entry of NMO-IgG into the CNS might establish a positive-feedback cycle of astrocyte damage, release and recruitment of inflammatory cells and mediators, and further breakdown of the blood-brain barrier. With regard to development of mouse models of NMO based on peripheral NMO-IgG administration, though serum antibody concentrations can be maintained, there is little or no CNS penetration of NMO-IgG in naïve mice, except for cirumventricular tissues or with various established maneuvers to permeabilize the blood-brain barrier.

Administration of NMO-IgG directly into brain by intraparenchymal infusion, which bypasses the blood-brain barrier, reproducibly produces NMO-like lesions when coinjected with human complement [20]. We found here that following single-site injection into brain parenchyma, NMO-IgG remained relatively localized in a 4.8 mm^2^ region around the injection site. Photobleaching measurement of FITC-dextran diffusion in brain suggest that molecules of IgG size are expected to diffuse throughout brain extracellular space [39]. The substantially restricted diffusion of NMO-IgG is the consequence of binding to astrocyte AQP4, as NMO-IgG diffusion was increased in brain of AQP4 knockout mice.

In conclusion, our studies establish pharmacological data on the processing of NMO-IgG in mice, including pharmacokinetics and tissue distribution. These data will facilitate the development of mouse models of NMO. The rapid and relatively unrestricted access of intravenously administered NMO-IgG to AQP4-expressing peripheral but not CNS tissues mandates the need for further investigation to explain the CNS specificity of NMO pathology and for development of creative strategies to promote access of NMO-IgG into the CNS to create animal models.

## Materials and Methods

### Mice

The generation of AQP4 null mice used in this study was described previously [12]. All experiments were performed on weight-matched wild type and AQP4 null mice on a CD1 genetic background, generally of age 16–18 weeks. Mice were maintained in air-filtered cages and fed normal mouse chow in the U.C.S.F Animal Care Facility. All procedures were approved by the U.C.S.F Committee on Animal research.

### NMO antibody, DNA constructs, cell culture and transfection

Purified human monoclonal NMO-IgG rAb-53 was generated as described [17], with a measles virus-specific rAb (2B4) used as an isotype-matched control [40]. Fluorescent labeling of NMO-IgG with Cy3 was done using the Amersham Cy3 Ab Labelling Kit (GE Healthcare, Buckinghamshire, UK). DNA constructs encoding full-length mouse AQP4 (M1 and M23 isoforms) were generated by PCR-amplification using whole brain cDNA as template. PCR fragments were ligated into mammalian expression plasmid pcDNA3.1 and sequenced. Chinese Hamster Ovary (CHO-K1) cells (ATCC CCL-61) were cultured at 37°C in 5% CO_2_/95% air in F12 Ham’s medium containing 10% fetal bovine serum (FBS) and 1% penicillin/streptomycin. Cells were grown on glass coverslips and transfected with plasmids in antibiotic-free medium using Lipofectamine 2000 (Invitrogen, Carlsbad, CA) according to the manufacturer’s protocol. Primary astrocyte cultures were generated from cortex of wild-type and AQP4-null neonatal mice, as described [41]. After 8-10 days in cultures, cells were treated for two weeks with 0.25 mM dibutyryl cAMP (Sigma-Aldrich, St. Louis, MO) to induce differentiation. Immunocytochemistry showed that > 95% of cells were positive for the astrocyte marker, glial fibrillary acidic protein (GFAP).

### Immunocytochemistry

AQP4-expressing cells were incubated for 20 min in blocking buffer (PBS containing 6 mM glucose, 1 mM pyruvate, 1% bovine serum albumin) and then for 30 min with NMO-IgG (20 µg/mL) in blocking buffer. Cells were then rinsed extensively with PBS, fixed in 4% paraformaldehyde for 15 min and permeabilized with 0.1% Triton X-100. Cells were then blocked again and incubated for 30 min with 0.4 µg/mL polyclonal, C-terminal-specific rabbit anti-AQP4 antibody (Santa Cruz Biotechnology, Santa Cruz, CA), then rinsed with PBS. Cells were then incubated for 30 min with 4 µg/mL goat anti-human IgG-conjugated Alexa Fluor 555 and goat anti-rabbit IgG-conjugated Alexa Fluor 488 (Invitrogen). Measurements of NMO-IgG/AQP4 binding affinity and kinetics were done as described [23].

### Pharmacokinetics

Wild-type or AQP4 null mice (weight-matched, 38–42 g) were injected by tail vein with 20 µg NMO-IgG or control IgG in 100 µL PBS. After 10 min, and 1, 24 and 48 hours, blood was collected through the tail vein, left for 30 min at room temperature to allow clotting of fibrinogen and platelets, and centrifuged for 15 min at 1500×g, 4°C. Serum was diluted 100-fold and NMO-IgG concentration was determined using a human IgG ELISA kit (GenWay, San Diego, CA). Serum from non-injected mice was used for background subtraction. Antibody half-life was calculated by linear regression on log_2_-transformed serum concentration as a function of time.

### Tissue distribution

Mice were injected with NMO-IgG as above. At 1 and 24 hours mice were anesthetized using 2,2,2-tribromoethanol (250 mg/kg intraperitoneally, Sigma-Aldrich) and perfused through the left cardiac ventricle with 20 mL PBS and then 10 mL of PBS containing 4% paraformaldehyde (PFA). AQP4-expressing tissues were removed, post-fixed 2 hours in 4% PFA and dehydrated overnight in 30% sucrose at 4°C. Tissues were embedded and frozen in OCT compound (Sakura Finetek, Torrance, CA) for sectioning and immunostaining. To label circumventricular organs in brain, 4% Evans Blue solution (200 µL) was injected by tail vein 2 hours before sacrifice. In some experiments pregnant mice (E18-19) were injected by tail vein with 50 µg NMO-IgG in 100 µL PBS. Mice were sacrificed 24 hours later and AQP4-expressing fetal tissues were processed as described above.

### Tissue immunofluorescence

Ten µm-thick frozen sections were blocked for 1 hour in PBS containing 10% goat serum (blocking buffer) and incubated for 1 hour at room temperature with 0.4 µg/mL rabbit anti-AQP4 antibody (Santa Cruz Biotechnology) diluted in blocking buffer. Anti-AQP4 antibody was detected using 4 µg/mL goat anti-rabbit IgG-conjugated Alexa Fluor 488 (Invitrogen). NMO-IgG (or control IgG) was detected using 4 µg/mL goat anti-human IgG-conjugated Alexa Fluor 555 (Invitrogen). Sections were then washed with PBS and mounted in Vectashield medium (Vector Laboratories, Burlingame, CA). Slides were imaged using a laser scanning confocal microscope D-Eclipse C1 (Nikon) equipped with a Nikon 100×, NA 1.49 Apo TIRF objective lens.

### Intracerebral injection of NMO-IgG

Mice were anesthetized as described above and mounted on a stereotactic frame. A midline scalp incision was made and a burr hole of diameter 1 mm was drilled in the skull 1 mm to the right and 1 mm posterior to bregma. A 30-gauge needle attached to 50-µL gas-tight glass syringe (Hamilton) was inserted 3 mm deep to infuse 3 µg NMO-IgG-Cy3 (or control antibody) in a volume of 10 µL (∼1 µL/min). At specified times after injection (3 mice per time point), mice were anesthetized and perfused through the left cardiac ventricle with 5 mL PBS and then 20 mL of PBS containing 4% PFA. Brains were post-fixed 2 hours in 4% PFA and processed for paraffin embedding. Five µm-thick sections were used for immunostaining. Areas of diffusion were quantified from fluorescence micrographs using Image J.

### Blood-brain barrier permeabilization

The blood-brain barrier was transiently permeabilized by intravenous injection of 250 ng pertussis toxin (from *Bordetella pertussis*, Sigma-Aldrich) in 100 µL PBS in the tail vein [31]. After 24 hours, an identical dose of toxin was administered together with 20 µg NMO-IgG in 100 µL PBS by tail vein injection. The blood-brain barrier was also permeabilized by intraperitoneal injection of LPS (*E. coli*, 055:B5, Sigma-Aldrich) at a dose of 3 mg/kg diluted in PBS to 0.3 mg/mL [32]. The LPS treatment was given twice, 48 hours apart, followed 24 hours later with NMO-IgG by tail vein injection. Control mice were injected with NMO-IgG in the same manner but received no pertussis toxin and no LPS (4 mice per group). Twenty-four hours following NMO-IgG administration, mice were injected intravenously with 4% Evans blue solution (160 mg/kg in PBS, Sigma-Aldrich) and sacrificed 2 hours later. At that time the left cardiac ventricle was perfused with 20 mL PBS. Half of the brain was fixed overnight at 4°C in 4% PFA and processed as described above for immunofluorescence. The other half of each tissue was weighed and immersed in 1 mL formamide (Sigma-Aldrich) at 55°C overnight to extract the Evans blue dye. Extracted dye was quantified by optical absorbance at 610 nm against Evans blue/formamide standards. Permeability of the blood-brain barrier to Evans blue was reported as µg of Evans blue per gram tissue. Spinal cord from these mice was also collected for immunofluorescence. Mechanical disruption of the blood-brain barrier in superficial brain cortex was accomplished by creating a burr hole in the skull using a high-speed drill as described earlier. NMO-IgG was injected into the tail vein 15 min before the surgery and mice were sacrificed 24 hours later.

### Statistical analysis

All data are presented as mean±S.E. The comparison of two groups in Fig. 2 and Fig. 4 was performed using unpaired two-tailed *t*-test.
